# Inhibitory Effects of LRP1-Based Immunotherapy on Cardiac Extracellular Matrix Biophysical Alterations Induced by Hypercholesterolemia

**DOI:** 10.1021/acs.jmedchem.2c02103

**Published:** 2023-04-28

**Authors:** Valerie Samouillan, Eduardo Garcia, Aleyda Benitez-Amaro, Maria Teresa La Chica Lhoëst, Jany Dandurand, Virginia Actis Dato, Jose Maria Guerra, Joan Carles Escolà-Gil, Gustavo Chiabrando, Carlos Enrich, Vicenta Llorente-Cortes

**Affiliations:** † CIRIMAT, Université de Toulouse, Université Paul Sabatier, Equipe PHYPOL, 31062 Toulouse, France; ‡ Biomedical Research Institute Sant Pau (IIB SANTPAU), Universitat Autonoma de Barcelona, 08041 Barcelona, Spain; § Institute of Biomedical Research of Barcelona (IIBB)-Spanish National Research Council (CSIC), 08036 Barcelona, Spain; ∥ Departamento de Bioquímica Clínica, Facultad de Ciencias Químicas, Universidad Nacional de Córdoba, X5000HUA Córdoba, Argentina; ⊥ Consejo Nacional de Investigaciones Científicas y Técnicas (CONICET), Centro de Investigaciones en Bioquímica Clínica e Inmunología (CIBICI), Godoy Cruz, 2290 Buenos Aires, Argentina; # Department of Cardiology, Hospital de la Santa Creu i Sant Pau, Biomedical Research Institute Sant Pau (IIB-SANTPAU), Universitat Autonoma de Barcelona, 08025 Barcelona, Spain; ∇ CIBERCV, Institute of Health Carlos III, 28029 Madrid, Spain; @ Metabolic Basis of Cardiovascular Risk, Biomedical Research Institute Sant Pau (IIB Sant Pau), 08041 Barcelona, Spain; ■ CIBER de Diabetes y enfermedades Metabólicas Asociadas (CIBERDEM), 28029 Madrid, Spain; ● Instituto Universitario de Ciencias Biomédicas de Córdoba (IUCBC), Centro de Investigación en Medicina Translacional Severo R. Amuchástegui (CIMETSA), G. V. al Instituto de Investigación Médica Mercedes y Martín Ferreyra (INIMEC-CONICET-UNC), X5016KEJ Córdoba, Argentina; ○ Unitat de Biologia Cellular, Departament de Biomedicina, Facultat de Medicina i Ciències de la Salut, Universitat de Barcelona, 08036 Barcelona, Spain; □ Centre de Recerca Biomèdica CELLEX, Institut d’Investigacions Biomèdiques August Pi i Sunyer (IDIBAPS), 08036 Barcelona, Spain

## Abstract

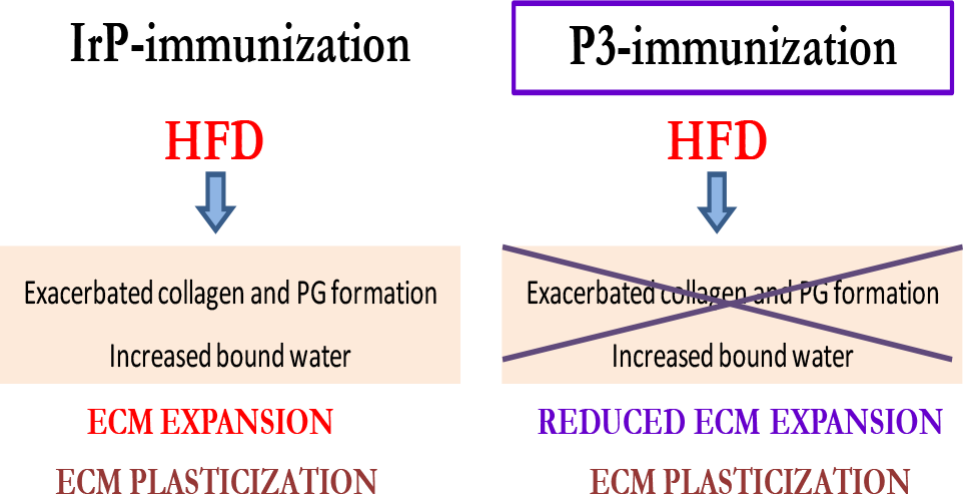

The accumulation of lipids in cardiomyocytes contributes to cardiac dysfunction. The specific blockage of cardiomyocyte cholesteryl ester (CE) loading by antibodies (Abs) against the P3 sequence (Gly^1127^–Cys^1140^) of the LRP1 receptor improves cardiac insulin sensitivity. The impact of anti-P3 Abs on high-fat diet (HFD)-induced cardiac extracellular matrix (ECM) biophysical alterations was analyzed. Both IrP (without Abs) and P3-immunized rabbits (with Abs) were randomized into groups fed either HFD or a standard chow diet. Cardiac lipids, proteins, and carbohydrates were characterized by Fourier transform infrared spectroscopy in the attenuated total reflectance mode. The hydric organization and physical structure were determined by differential scanning calorimetry. HFD increased the levels of esterified lipids, collagen, and α-helical structures and upregulated fibrosis, bound water, and ECM plasticization in the heart. The inhibitory effect of anti-P3 Abs on cardiac CE accumulation was sufficient to reduce the collagen-filled extracellular space, the level of fibrosis, and the amount of bound water but did not counteract ECM plasticization in the heart of hypercholesterolemic rabbits.

## Introduction

Heart failure is a progressive and lethal disorder, and its prevalence has increased steadily in developed countries and will probably continue to increase in the coming years.^1^ Management of this disease has focused mainly on the control of risk factors, such as hypertension and ischemic heart disease, which are the main causes of heart failure.^2^

A recognized cardiovascular risk factor is low-density lipoprotein (LDL) cholesterol, which has been unequivocally established as one of the main risk factors in ischemic heart disease due to its causal involvement in atherosclerosis plaque formation and progression.^3,4^ However, the direct impact of lipoproteins on the myocardium has been scarcely explored in the clinical setting despite consistent experimental evidence supporting the crucial role of cholesteryl ester (CE)-enriched lipoproteins in cardiac dysfunction.

In humans, hyperlipidemia has been reported to cause the accumulation of lipid into cardiomyocytes, altering cardiac function through the induction of electrophysiological changes in the heart.^5,6^ LDL cholesterol levels have been reported to negatively correlate with endocardial longitudinal strain and circumferential strain in hypercholesterolemia familiar patients.^7^ Moreover, LDL has been causally related with left ventricle (LV) dysfunction in a Mendelian randomization study using instrumental variable analysis in 17 311 European individuals with paired genotype and cardiac magnetic resonance data and subsequent sensitivity analysis using summary-level data.^8^ In addition, hyperlipidemia has been reported to enhance LV hypertrophy in diabetic patients.^9^

Experimental studies have shown that lipoproteins from the diet are an essential source of fatty acids for heart mitochondria.^10,11^ The problem is that CE-enriched lipoproteins, both very-low-density lipoprotein (VLDL) and low-density lipoproteins (LDLs), also contribute to intracellular accumulation of CE in cardiomyocytes, causing serious alterations in sarco(endo)plasmic reticulum calcium ATPase-2 (SERCA 2) and calcium metabolism.^12−15^*In vivo*, hypercholesterolemic diets enrich the sarcoplasmic reticulum (SR) with cholesterol, promoting ventricular vulnerability to fibrillation and systolic and diastolic dysfunction in rabbits.^16,17^ Biophysical studies allowed us to identify the spectral lipid Fourier transform infrared (FTIR) signatures of circulating LDL and VLDL in the heart of hypercholesterolemic rabbits.^18^ These results highlight the crucial effects of hypercholesterolemia and CE-enriched lipoproteins in the electrical and metabolic remodeling of the heart.

Ventricular dysfunction in patients with heart failure is associated with cardiac fibrosis, an excessive deposition of extracellular matrix (ECM) proteins. Changes in ECM composition and ultrastructure define alterations in myocardium architecture that cause arrhythmias and cardiac dysfunction.^19−21^ Cardiac fibrosis is a common feature in hypertrophic, dilated, restrictive, and inflammatory cardiomyopathies and underlies the pathogenesis of both heart failure with a reduced ejection fraction and heart failure with a preserved ejection fraction.^22,23^

The main ECM components responsible for maintaining the biochemical and biophysical properties of the ECM are fibrillar collagen, elastin, and proteoglycans (PGs). ECM in the heart is primarily composed of collagen type I (85%) and type III (11%) and other less abundant collagens, including types IV–VI.^24^ The main modulators of ECM metabolism and functionality in heart failure are mechanosensitive pathways and neurohumoral mediators regulating growth factor secretion by cardiomyocytes, vascular cells, and immune cells and pressure interstitial overloading that alters the protease/antiprotease balance promoting dilatation, cardiac remodeling, and systolic dysfunction.^19,20^ Our unprecedented results recently showed a crucial additional factor for ECM remodeling consisting of CE loading of smooth muscle cells^25^ and cardiomyocytes.^26^ CE loading of cardiac cells causes dramatic changes in the physical structure of secreted tropoelastin. These results open the possibility of using low-density lipoprotein receptor-related protein 1(LRP1)-based approaches inhibiting cardiomyocyte CE loading,^12−14^ as a potential strategy for modulating pathological ventricular remodeling in metabolic diseases.

LRP1 is a key receptor involved in the selective uptake of CE from CE-enriched lipoproteins in vascular cells.^27,28^ Through molecular, structural, and proteomic studies, we determined one specific LRP1 sequence that interacts with ApoB100.^29,30^ On the basis of this information, we developed an LRP1 (P3)-based immunotherapy with specificity and selectively to inhibit the accumulation of CE in vascular smooth and cardiac muscle cells *in vivo* in a rabbit model of hypercholesterolemia.^18,31^ This immunotherapy increases the serum levels of anti-P3 Abs that efficiently compete with CE-enriched lipoproteins inhibiting atherosclerosis in vasculature^31^ and increasing insulin sensitivity in the heart of the rabbit model.^18^ This unique rabbit model offers the possibility of testing the pathological and specific impact of intracellular CE accumulation in processes related to cardiac functionality such as extracellular matrix composition, fibrosis, and plasticization. On the basis of these previous results, the aim of this study is to analyze the effect of myocardial CE accumulation on the biophysical ECM changes induced by a high-fat diet (HFD) in the heart. For that, we specifically blocked the interaction of CE-enriched lipoproteins with the LRP1 receptor via immunization with the P3 peptide.

## Results and Discussion

### Pattern of FITR Bands in the Myocardium of Rabbits

FTIR provides information about metabolic (lipid bands) and structural (ECM bands) components in an integrated and unique spectrum from each analyzed sample.^32^ Imaging and data analysis (computed and processed) are directly performed on tissues without complex preparation, ensuring the maintenance of chemical structures. The spectral signature of the heart of rabbits (Table S1) is like that reported for rat,^33,34^ mouse,^35,36^ pig,^37^ and human^38,39^ hearts.

The major absorption bands in these spectra are the amide A (3282 cm^–1^), amide I (1646–1636 cm^–1^), and amide II (1538 cm^–1^) bands, mainly associated with proteins in freeze-dried tissues, and the CH*x* stretching zone (3000–2800 cm^–1^) related to the whole lipid part.

The major ventricular proteins are cardiomyocyte myofibrillar proteins (myosin and α-actin), sarcoplasmic proteins, and the main structural proteins of the ECM, such as fibrillary collagens I and III. It is not possible to discriminate between the different proteins from these spectra, except between collagens I and III, which possess a specific quadruplet at 1334, 1280, 1234, and 1202 cm^–1^.^33,35,36,40^ In addition to lipids and proteins, other components of the heart tissue participate to a low or moderate degree in the vibrational signature in the range of 1800–1000 cm^–1^, and, in particular, proteoglycans (PG) and carbohydrates.^32−34^

### Biophysical Validation of the Efficacy of Anti-P3 Abs to Reduce the Level of Accumulation of Myocardial CE

**Figure 1:**
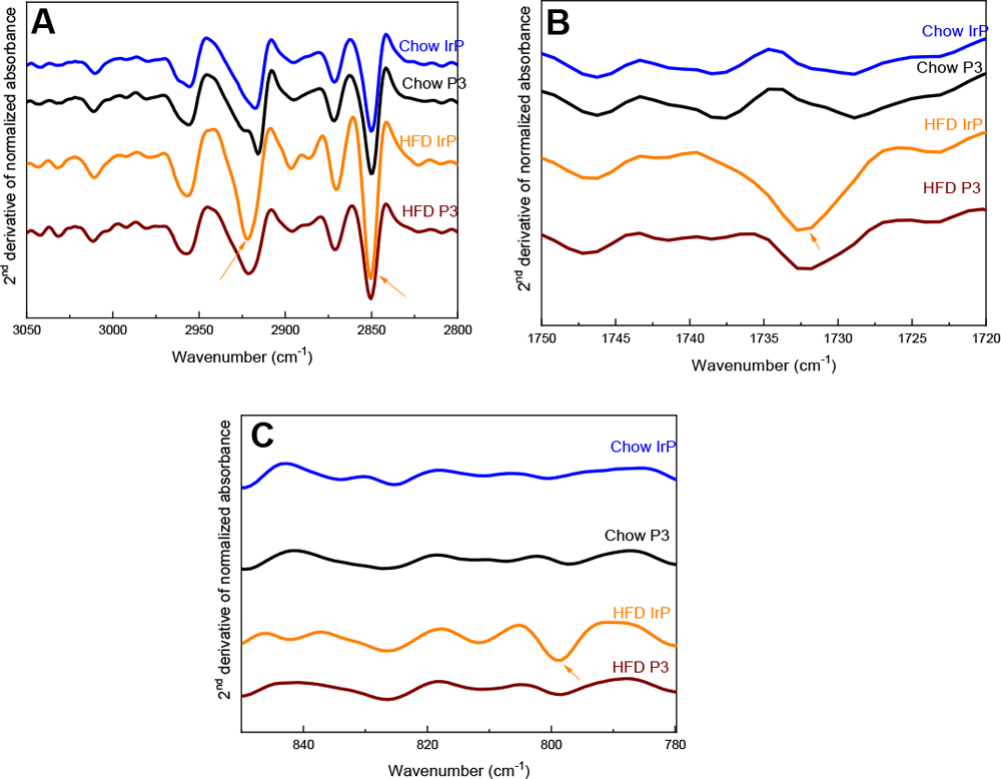
FTIR-ATR second-derivative mean spectra of rabbit hearts in (A) the specific CH*x* stretching zone, (B) the specific C=O ester stretching zone, and (C) the specific C–H CE deformation zone. Arrows indicate the increase in (A) the CH_2_ stretching bands of lipids (2922 and 2851 cm^–1^), (B) the C=O stretching band of esterified lipids (1732 cm^–1^), and (C) the C–H bending of cholesteryl esters (800 cm^–1^) specifically in heart from the HFD/IrP group but not from the other groups. *n* = 5 per group.

Here, FTIR lipid spectra of the heart showed that absorption bands in the range of 3050–2800 cm^–1^ are mainly associated with the intense CH_2_ stretching of long hydrocarbon chains, accounting for the whole lipid part (Figure 1A). The specific absorption of C=O stretching from esterified lipids^33^ and the specific CE absorption^41^ are present in the 1745–1735 cm^–1^ (Figure 1B) and 800 cm^–1^ (Figure 1C) zones of the cardiac spectra. These bands were detected irrespective of the diet or treatment at similar wavenumbers, indicating that the nature of components is identical in the hearts of the different groups.

In line with previous chromatographic results,^18^ the intensities of these specific lipid absorption bands strongly increased in the hearts of HFD-fed control rabbits (HFD/IrP) (Figure S1A,B, arrows), but not in P3-immunized rabbit hearts.

**Figure 2:**
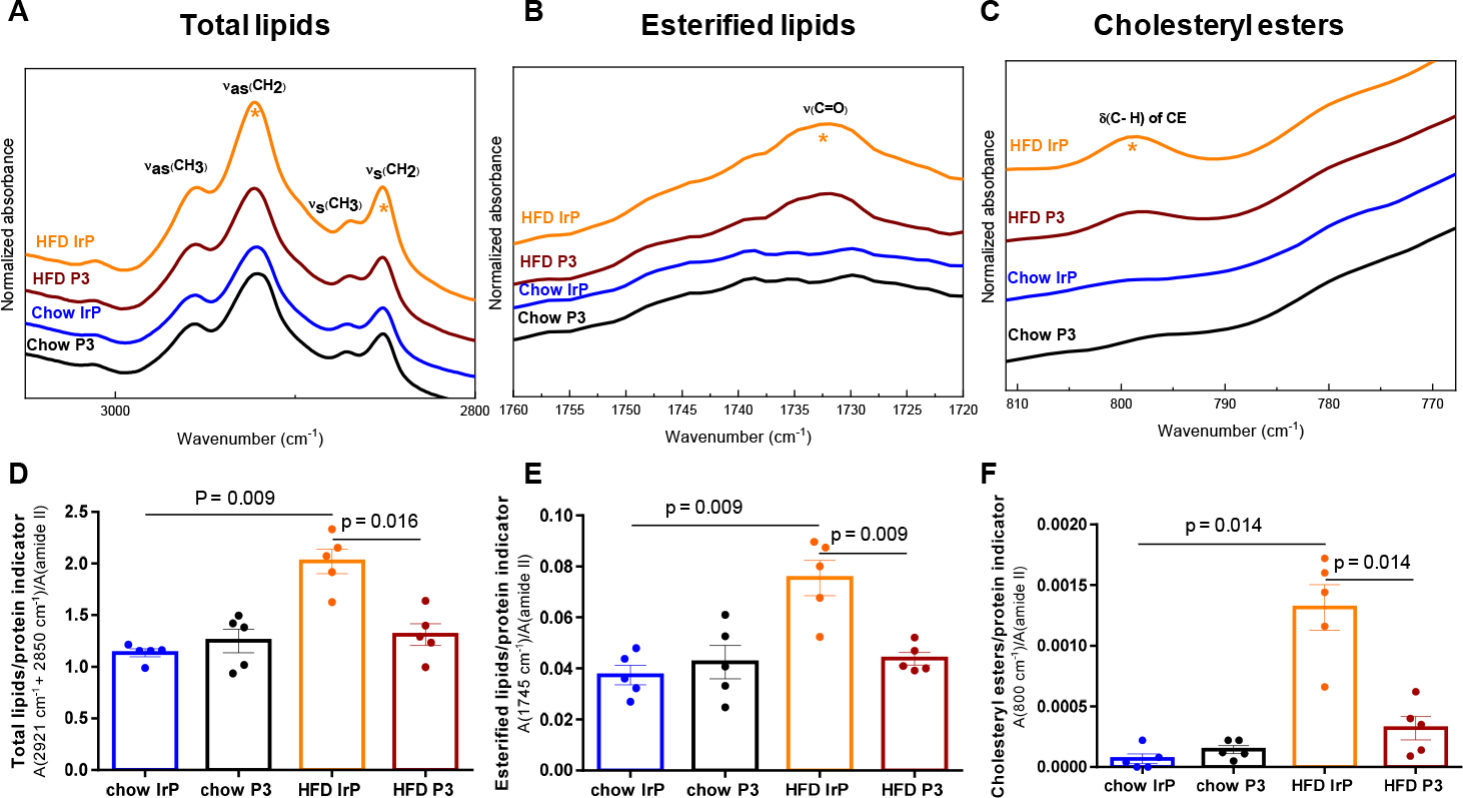
Infrared analysis reflects the massive amount of accumulation of lipid in HFD/IrP rabbit hearts. (A–C) Normalized mean FTIR-ATR spectra of rabbit heart in the CH*x* stretching zone, the C=O stretching zone of esterified lipids, and the C–H deformation zone of cholesteryl esters, respectively. Asterisks indicate the intensity bands that are increased in the hearts from HFD/IrP rabbits compared to the other rabbit groups. Bar graphs showing the results from quantitative analysis of spectra for (D) total lipids, (E) esterified lipids, and (F) cholesteryl esters. *n* = 5 per group. Results are shown as means ± the standard deviation (SD). The statistical significance was determined by a Mann–Whitney U nonparametric test.

This striking difference in the lipid content of the HFD/IrP group compared to that of the other three groups is more clearly highlighted on the second-derivative spectra of HFD/IrP rabbit hearts, indicative of the enhancement of the signatures of total lipids (Figure 1A, arrows), esterified lipids (Figure 1B, arrow), and CE (Figure 1C, arrow) in this group. The quantitative analysis from individual heart spectra showed a significant increase for the total lipid/protein ratio {area (2921 + 2850 cm^–1^)/[A(amide II)]} (Figure 2A,D), the massive accumulation of esterified lipids {area (1745 cm^–1^)/[A(amide II)]} (Figure 2B,E), and in particular CE {area (800 cm^–1^)/[A(amide II)]} (Figure 2C,F). These studies also showed the high efficacy of anti-P3 Abs to inhibit cardiac lipid and, in particular, cardiac CE loading.

In addition, the level of CE with phospholipids (PLs) {area (1171 cm^–1^)/[A(amide II)]} (Figure S2A) in hearts of control HFD-fed rabbits was also drastically reduced in the heart of P3-immunized (HFD/P3) rabbits (Figure S2B).

As expected, HMG-CoA reductase (HMGCoAR) and LDLR levels were significantly reduced in the heart of hypercholesterolemic rabbits (Figure S3). The levels of cholesterol 7α-hydroxylase (CYP7A1) were almost undetectable in the heart of this experimental model (data not shown).

The high level of dispersion of points in each group may be related to the high cell variability between closed heart layers and closed heart zones previously reported in complex tissues such as the heart.^42,43^ In fact, our previous biochemical quantification of cardiac cholesteryl esters through thin layer chromatography^18^ and our current results from real time polymerase chain reaction (PCR) analysis also showed a similar dispersion of data points.

Principal component analysis (PCA) of the FTIR spectra allows a decrease in the large number of variables (wavenumbers) to a new set of uncorrelated small numbers of variables, called the principal components (PCs). PCA allows the graphical representation of each spectrum as a scatter plot in the new base of PCs (Figure S4A). In this representation, the discrimination of HFD/IrP rabbit hearts from the other three types of rabbit hearts was clearly highlighted. It is noteworthy that the spectra of HFD/IrP rabbit hearts were discriminated by negative coordinates on the PC1 axis, which contributes 53% of the variance (Figure S4B). The major negative contributions of the PC1 axis were found at 2925 and 2853 cm^–1^ (total lipids), 1737 cm^–1^ (esterified lipids), 1465 cm^–1^ (total lipids), 1172 cm^–1^ (CE and phospholipids), and 1080–1050 cm^–1^ (carbohydrate residues, polysaccharides, and collagens of the ECM). Because the coordinates of the spectra of HFD/IrP rabbit hearts along the PC1 are negative (in contrast to the other three categories of spectra of rabbit hearts), this analysis corroborates that the differential evolution of the spectral signature of the HFD/IrP group compared to the other groups was mainly due to the lipid phase and, in particular, to CE. In addition, PCA also suggests that ECM components (mainly collagen and polysaccharides, associated with 1080–1050 cm^–1^ absorption) contribute to the differential spectral signature of HFD/IrP hearts.

Taken together, these results highlight the strong upregulatory effect of the HFD diet on the accumulation of neutral lipids in the hearts of rabbits and the efficacy of anti-P3 Abs to reduce myocardial CE accumulation in line with previous lipidomic and imaging results in this model.^18^ Cardiomyocytes are the major cell components of the adult heart (49.2%),^44^ and they have the capacity to produce and secrete ECM components, including collagen and PGs.^45^ Our results from this translational rabbit model suggest that the cardiomyocyte LD-CE content may influence the ECM composition. Therefore, we explored in this model whether anti-P3 Abs, with specifically reduce the level of accumulation of intracellular CE in rabbit cardiomyocytes,^18^ reverse HFD-induced biophysical ECM alterations in the heart.

### Effect of Anti-P3 Abs on HFD-Induced Changes in the Biophysical ECM Structure and Composition

**Figure 3:**
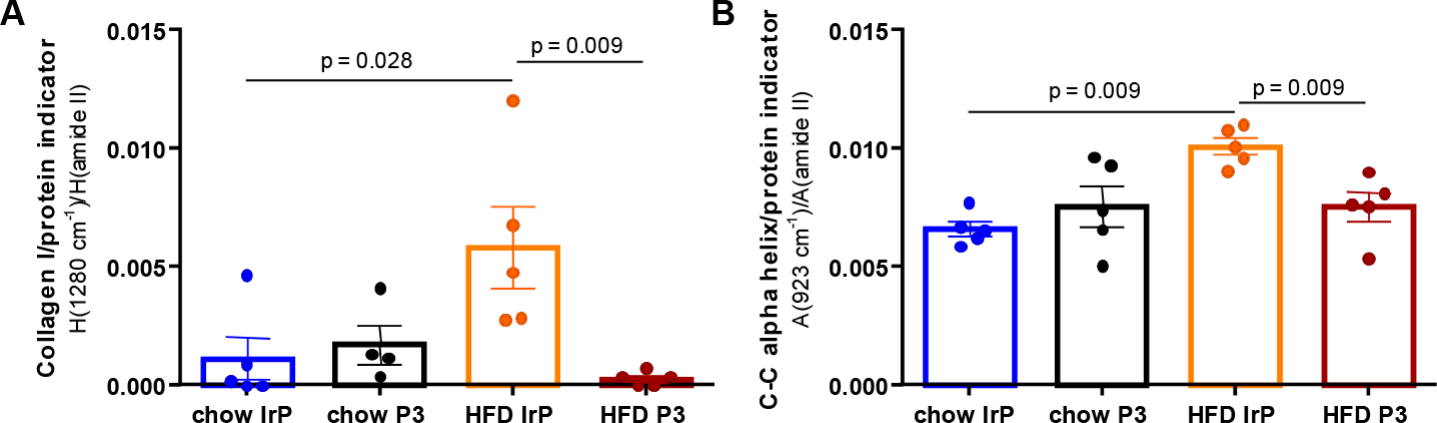
Infrared analysis reflects the increase in collagen and C–C vibrations in α-helical proteins in HFD/IrP rabbit hearts. Bar graphs show the results from quantitative analysis of spectra for (A) the height of the 1280 cm^–1^ band corresponding to collagen and (B) the area of the 923 cm^–1^ band assigned to C–C α-helical structures, both normalized to the 1540 cm^–1^ band (height or area) assigned to total proteins. *n* = 5 per group. Results are shown as mean ± SD. Statistical significance was determined by a Mann–Whitney U nonparametric test.

The comparison of the second-derivative spectra of rabbit hearts in the amide III zone (1300–1200 cm^–1^) (Figure S5) (where absorption bands from collagen, nucleic acids, phospholipids, and proteoglycans overlap) revealed the intensification of the 1280 cm^–1^ band of collagen^32,33,36,40^ and the 1234 and 1226 cm^–1^ bands corresponding to collagen^33^ and PGs^34^ in HFD/IrP rabbit hearts (Figure S5, arrows). Due to the overlapping of these bands, PG quantification from individual spectra was not feasible. Strikingly, anti-P3 Abs inhibited the increase in the intensities of several FTIR bands induced by HFD in the heart of rabbits such as the 1280 cm^–1^ band associated with collagen^33,36^ (Figure 3A), the 923 cm^–1^ band associated with α-helical structures^49^ (Figure 3B and Figure S5), and the 1234 and 1226 cm^–1^ bands corresponding to collagen^33,36^ and PGs^33,34^ (Figure S5). The increase in the level of PG observed in the heart of HFD/IrP rabbits is in line with the upregulatory effects of intracellular cholesterol on proteoglycan production in smooth muscle and endothelial cells.^46,47^ The membrane cholesterol composition also modulates the activity of the hyaluronan synthase, a membrane-associated glycosyltransferase that modulates glycosaminoglycan structure.^48^ These results point to HFD as a key inducer of alterations in ECM composition and structure.

In line, the associated collagen/protein indicator (1280 cm^–1^ band/amide II height ratio) was significantly increased by HFD in HFD/IrP but not in HFD/P3 rabbit hearts (Figure 3A).

Exposure to a HFD also increased the intensity of the 922–930 cm^–1^ band, corresponding to C–C vibrations in α-helical proteins^49^ and quantified via the associated indicator only in HFD/IrP hearts (Figure 3B).

In agreement, the increased level of α-helical proteins was observed in the second-derivative spectra of HFD/IrP (Figure S6, arrow) but not in that of HFD/P3 hearts.

As opposed to the intensification of the 1280 and 1234 cm^–1^ bands assigned to collagen,^33,36,40^ a decrease in the intensity of the 1643 cm^–1^ band of the second-derivative spectra (corresponding to random coil or β-sheets) was detected in the hearts of HFD/IrP rabbits (Figure S7, asterisks). Anti-P3 Abs counteracted the decrease in the intensity of the 1643 cm^–1^ band caused by HFD (Figure S7).

The amide I band, which is mainly due to C=O stretching of proteins, is very sensitive to secondary structures in proteins.^33^ However, if some regions of amide I are unambiguously attributable to specific secondary structures in the case of pure proteins, this remains difficult in the case of freeze-dried biological tissues, especially for the component at 1643 cm^–1^ in the border zone. According to literature data, it could be classified as either β-sheets^50^ or random coil,^51^ and it is a minor component of pure collagen.^52,53^ The decrease in the level of this type of structure could be due to the increase in the level of collagen compared to other proteins. It is noteworthy that a reduction in the level of this type of secondary structures in the ECM, concomitant with the increase in the level of cardiac collagen, was previously reported by our team in the heart of diabetic rats.^26^ Remarkably, anti-P3 Abs counteracted the decrease in the intensity of the 1643 cm^–1^ band (corresponding to random coil or β-sheets) caused by HFD (Figure S7). Together, our results point to HFD and in particular to CE-enriched lipoproteins, as a key inducer of alterations in ECM composition and structure.

### Effect of Anti-P3 Abs on HFD-Induced Cardiac Fibrosis

**Figure 4:**
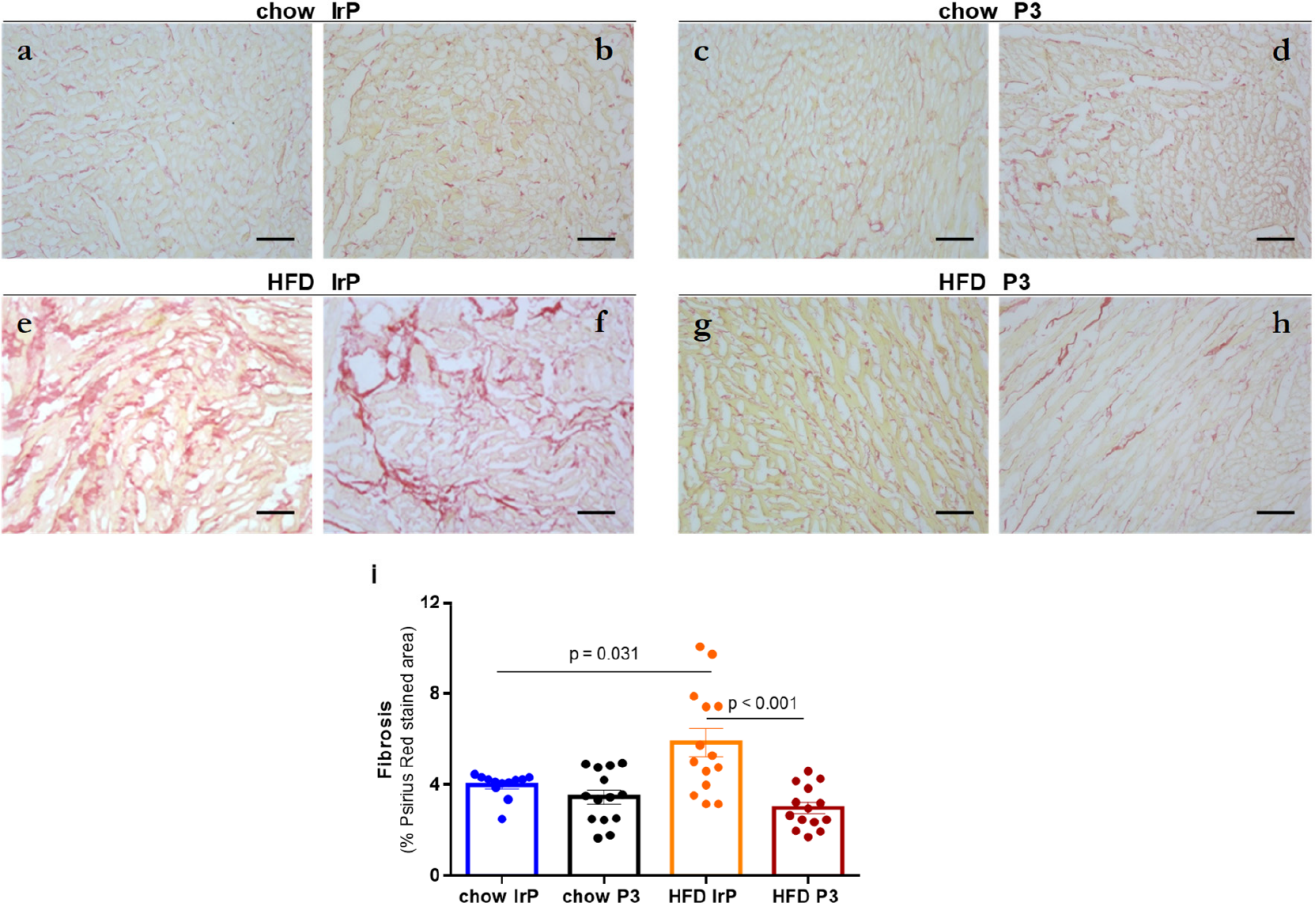
Immunohistochemical analysis showing the increased frequency of cystic fibrosis of HFD/IrP rabbit hearts. Representative immunohistochemical images showing Psirius red stained areas in the rabbit heart sections of (a and b) chow/IrP, (c and d) chow/P3, (e and f) HFD/IrP, and (g and h) HFD/P3 and (i) bar graphs showing the percentage of Psirius red-positive areas normalized to the total area of the cardiac section: Chow/IrP (*n* = 12 sections), chow/P3 (*n* = 15 sections), HFD/IrP (*n* = 17 sections), and HFD/P (*n* = 16 sections). Results are shown as means ± SD. Statistical significance was determined by one-way analysis of variance with Tukey’s posthoc test. The bar size is 10 μm.

In agreement with biophysical results, immunohistochemical studies showed a strong increase in the frequency of cardiac fibrosis (measured as Psirius red staining) induced by HFD (Figure 4). These results are in good agreement with results from other *in vivo* studies supporting the impact of HFD per se on interstitial myocardial fibrosis in mice^54,55^ and obese minipigs.^56^ Anti-P3 Abs were highly efficient in decreasing the frequency of HFD-induced cardiac fibrosis because Psirius red staining was almost absent in HFD/P3 hearts (Figure 4g–i), like in chow/IrP hearts (Figure 4a,b,i) and chow/P3 heart (Figure 4c,d,i), as opposed to the intense staining of HFD/IrP hearts (Figure 4e,f,i).

**Figure 5:**
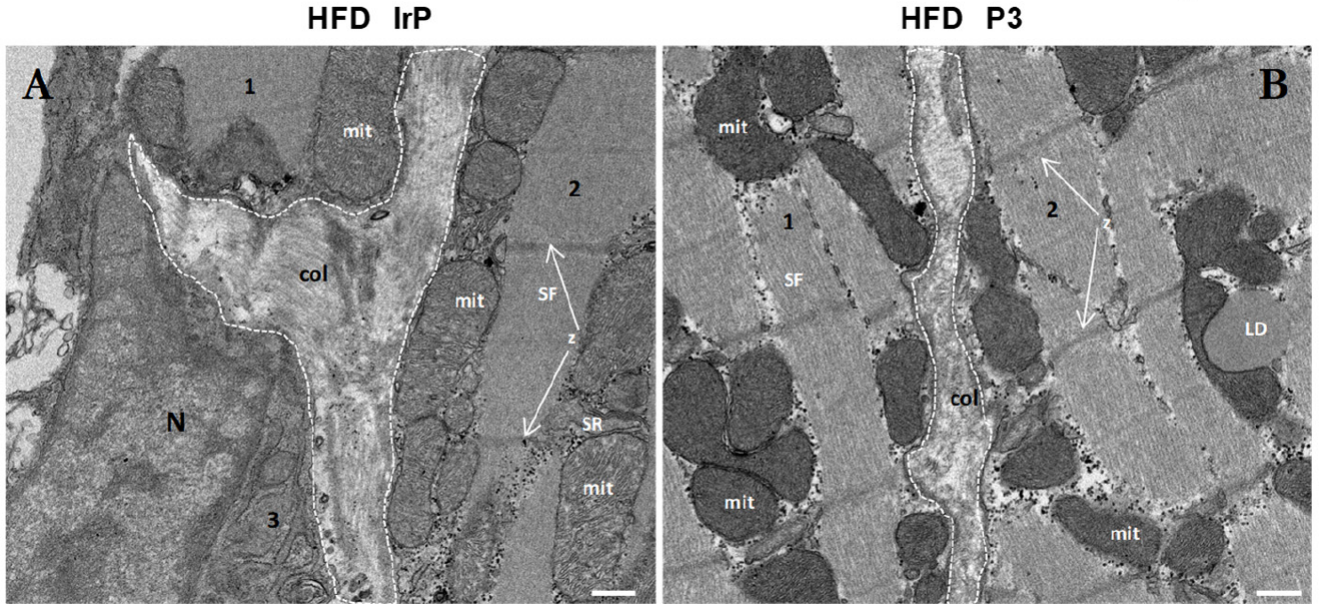
Anti-P3 Abs reduce the level of HFD-induced ECM deposition in the heart. Representative transmission electron microscopy (TEM) images of cardiac intracellular and extracellular ultrastructure showing differences between ECM deposition and collagen organization in the heart of HFD/IrP and HFD/P3 rabbit groups. Mitochondria (mit) in the intersarcomeric cytoplasm between the myofibrils (MF) are closely connected with sarcoplasmic reticulum (SR). (A) In the HFD/IrP heart, an increased ECM (highlighted by dashed white points) occupied by collagen is in contact with three cells (1–3). (B) In the HFD/P3 heart, a less-expanded ECM is occupied by disordered collagen fibers that are in contact with two cardiomyocytes (1 and 2), one of them containing a large and electrodense lipid droplet (LD) in its cytoplasm. Representative Z-discs (Z) are indicated. Numbers refer to the different cells in the section. The scale bar is 400 nm.

In agreement with biophysical and immunohistochemical results, transmission electron microscopy (TEM) of ultrathin sections of the heart revealed a narrowing of the cardiac ECM in HFD/P3 rabbits (Figure 5B) as compared to HFD/IrP rabbits (Figure 5A). Together, these results support that myocardial CE accumulation plays a key role in HFD-induced alterations in ECM composition and structure.

### Effect of HFD in the Water Content of the Heart in the Absence and Presence of Anti-P3 Abs

**Figure 6:**
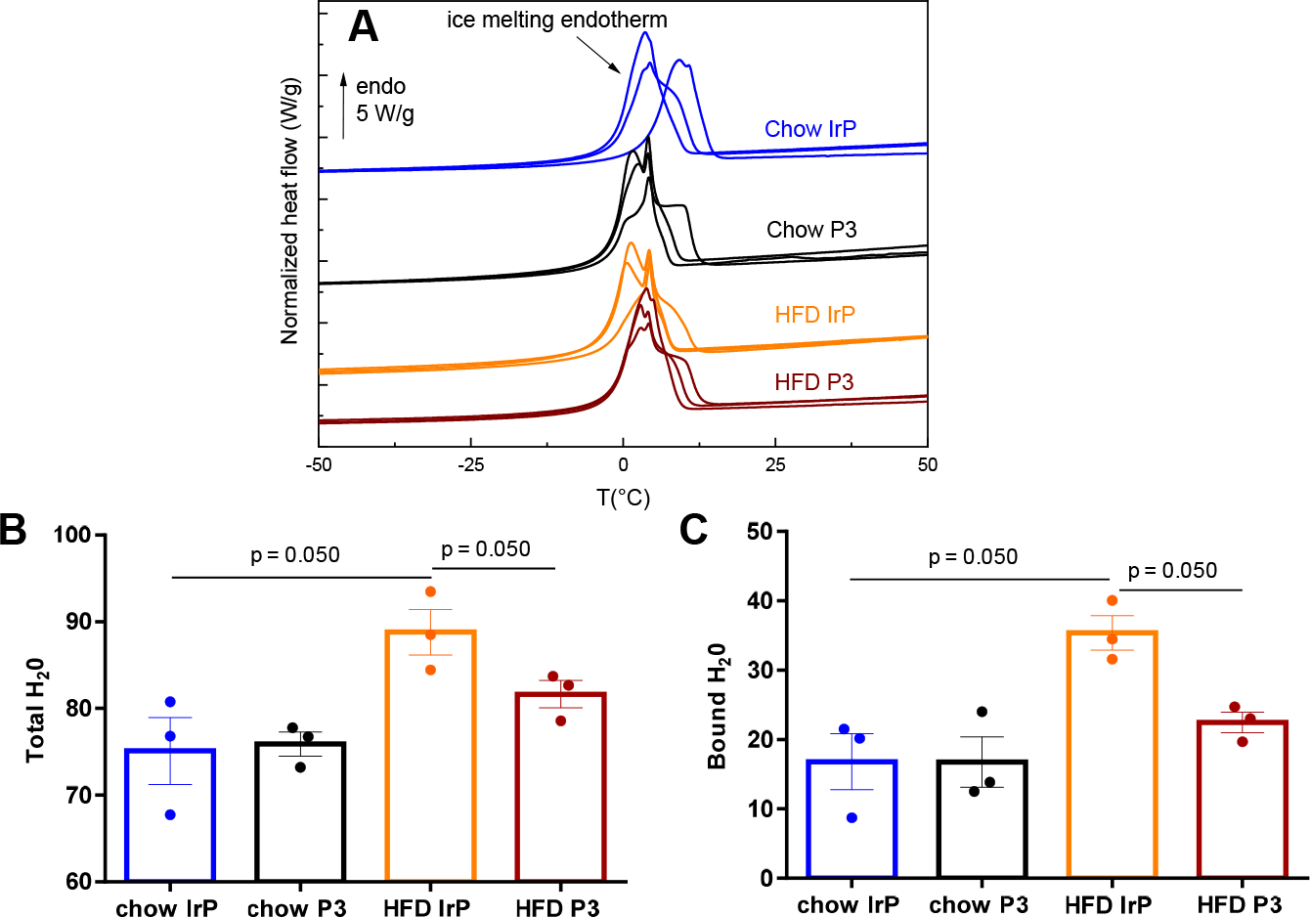
Differential scanning calorimetry (DSC) analysis showing the increased total and bound water content of HFD/IrP rabbit hearts. (A) Representative DSC thermograms of fresh rabbit hearts in the range of −50 to 50 °C. Bar graphs show (B) total or (C) bound water (grams of water per gram of tissue) in the heart. *n* = 3 per group. Results are shown as means ± SD. Statistical significance was determined by a Mann–Whitney U nonparametric test.

DSC thermograms of initially frozen rabbit hearts are characterized by a main endothermic peak (between −10 and 10 °C), attributed to the melting of ice (Figure 6A), as in most hydrated biological tissues. The area of this peak can be used to quantify the amount of freezable water,^57^ namely bulk or free water in the tissue. After a complete dehydration, the total amount of hydration of the sample can be determined and, by difference, the amount of bound water. HFD significantly increased the percentage of total water (accounting for more than three-quarters of the weight in the wet heart) (Figure 6A,B) and bound water (accounting for 17% by grams of water per gram of wet tissue) in hearts of HFD/IrP (Figure 6A,C). The percentage of bound water is a minor part in the heart of chow-fed rabbits, in agreement with previous results in non-ischemic hearts from control mice^35^ and humans.^39^ Anti-P3 Abs efficiently reduced the percentage of total (Figure 6A,B) and bound water (Figure 6A,C) in hypercholesterolemic rabbits. There were no differences in the cardiac free water content between groups (data not shown).

These results indicate that the increase in the total number of waters induced by HFD in the heart of rabbits is mainly due to the increase in the number of bound waters and that P3 immunization prevents the upregulatory effects of HFD on the number of total and bound waters in the heart. The capacity of PGs to retain and increase the bound water content in the ECM of several tissues^58,59^ suggests that the higher bound water content in HFD/IrP hearts is related to their higher collagen/proteoglycan content. It is noteworthy that P3 immunotherapy had a high efficacy at blocking the HFD-induced collagen/proteoglycan content as well as reducing the bound water content in the ECM of the heart. Together, these results indicate that the increased total water levels induced by HFD in the heart of rabbits are mainly due to the increase in PG-bound water content, and that both total and bound water content increases are dependent on myocardial CE content.

### Effect of HFD and Anti-P3 Abs on Cardiac Tissue Plasticization

**Figure 7:**
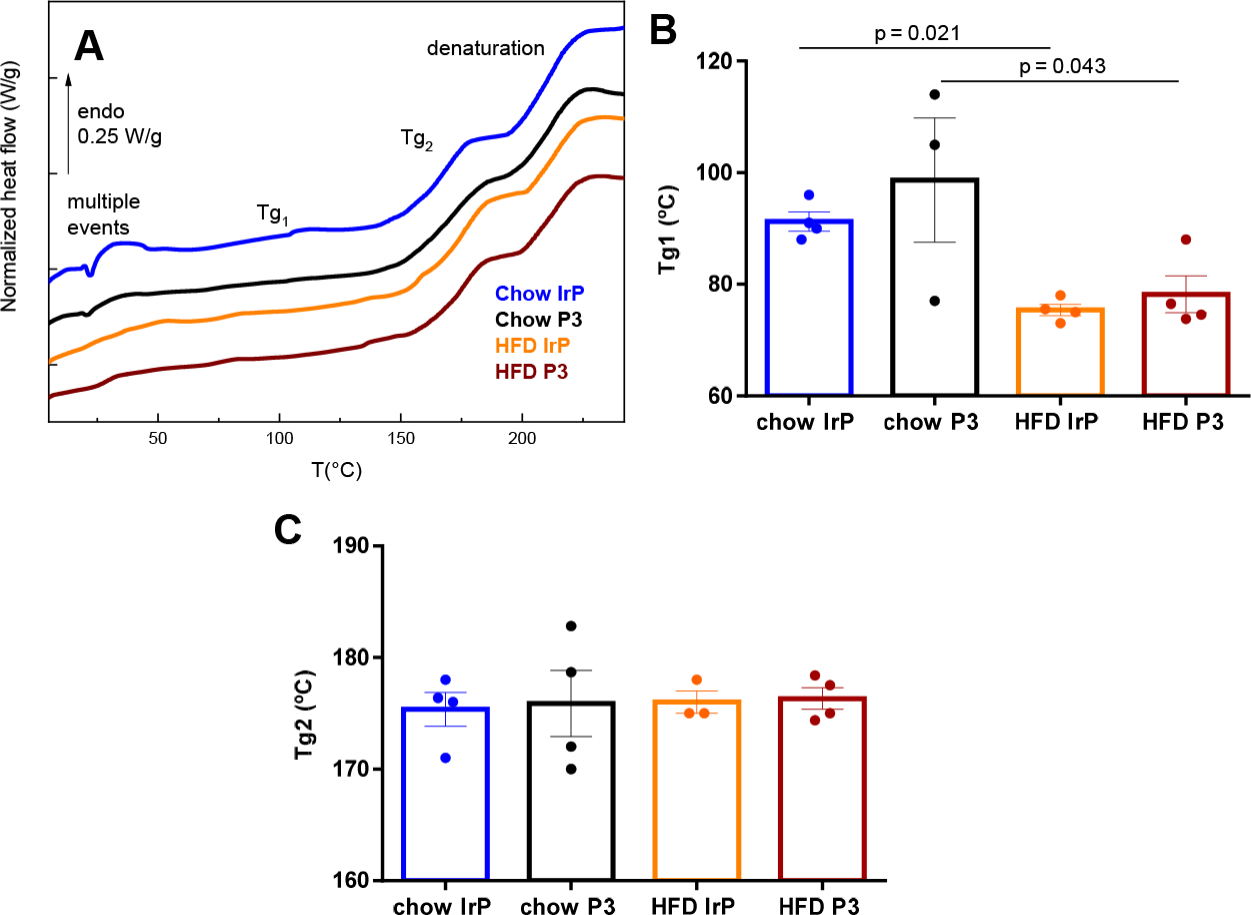
Differential scanning calorimetry (DSC) analysis showing the plasticization of the soft amorphous phase of heart from HFD-fed rabbits. (A) Representative DSC thermograms of freeze-dried rabbit heart in the range of 15–230 °C. Bar graphs show (B) glass transition temperature *T*_g1_ and (C) glass transition temperature *T*_g2_. *n* = 4 per group. Results are shown as means ± SD. Statistical significance was determined by a Mann–Whitney U nonparametric test.

DSC thermograms of freeze-dried samples showed several transitions that include multiple endothermic peaks in the range of 0–60 °C associated with the lipid phase (Figure 7A). These thermograms evidenced a first detectable glass transition (temperature *T*_g1_) (reversible on successive scans) between 50 and 110 °C and a second glass transition (temperature *T*_g2_) (reversible on successive scans) between 160 and 200 °C. The occurrence of two glass transitions is indicative of the presence of amorphous phases of different softness in the heart tissues undergoing their transition from a glassy to a rubber state during heating. These complex amorphous phases are constituted by proteins that do not possess long-range order, such as elastin,^25,60−62^ but also by ground substance or amorphous material mainly composed of PGs and glycosaminoglycans (GAGs). Thermal denaturation of the protein phase (irreversible on successive scans) was detected above 200 °C and was associated with the thermal unfolding of long-range order proteins (e.g., collagens, myosin, and actin). Note that the different transitions (glass transitions and denaturation) are shifted toward high temperatures, as tissues are analyzed in the freeze-dried state to avoid the predominant answer of water. These DSC measurements indicate that the glass transition temperature, *T*_g1_, was drastically decreased (by >15 °C) in the heart of HFD-fed rabbits, independent of the absence or presence of anti-P3 Abs (Figure 7A,B). In contrast, there were no differences between groups for glass transition temperature *T*_g2_ (Figure 7A,C) or for the denaturation temperature and associated area (data not shown).

The reductions in glass transition temperature *T*_g1_ (ascribed to the softer phase) in the heart of HFD/IrP and HFD/P3 groups are indicative of cardiac plasticization. Plasticization of macromolecules, the depression of the glass transition, needs the presence of plasticizers, which are effective in disrupting intermolecular interactions and increasing the free volume and therefore the segmental mobility of the chains.^63^ Water is known as an excellent plasticizer of biomacromolecules,^63,64^ and the increase in the bound water content in HFD/IrP tissues could explain at first glance this phenomenon; nevertheless, plasticization was not reversed in the heart of HFD/P3 rabbits, which have a smaller amount of bound water.

**Figure 8:**
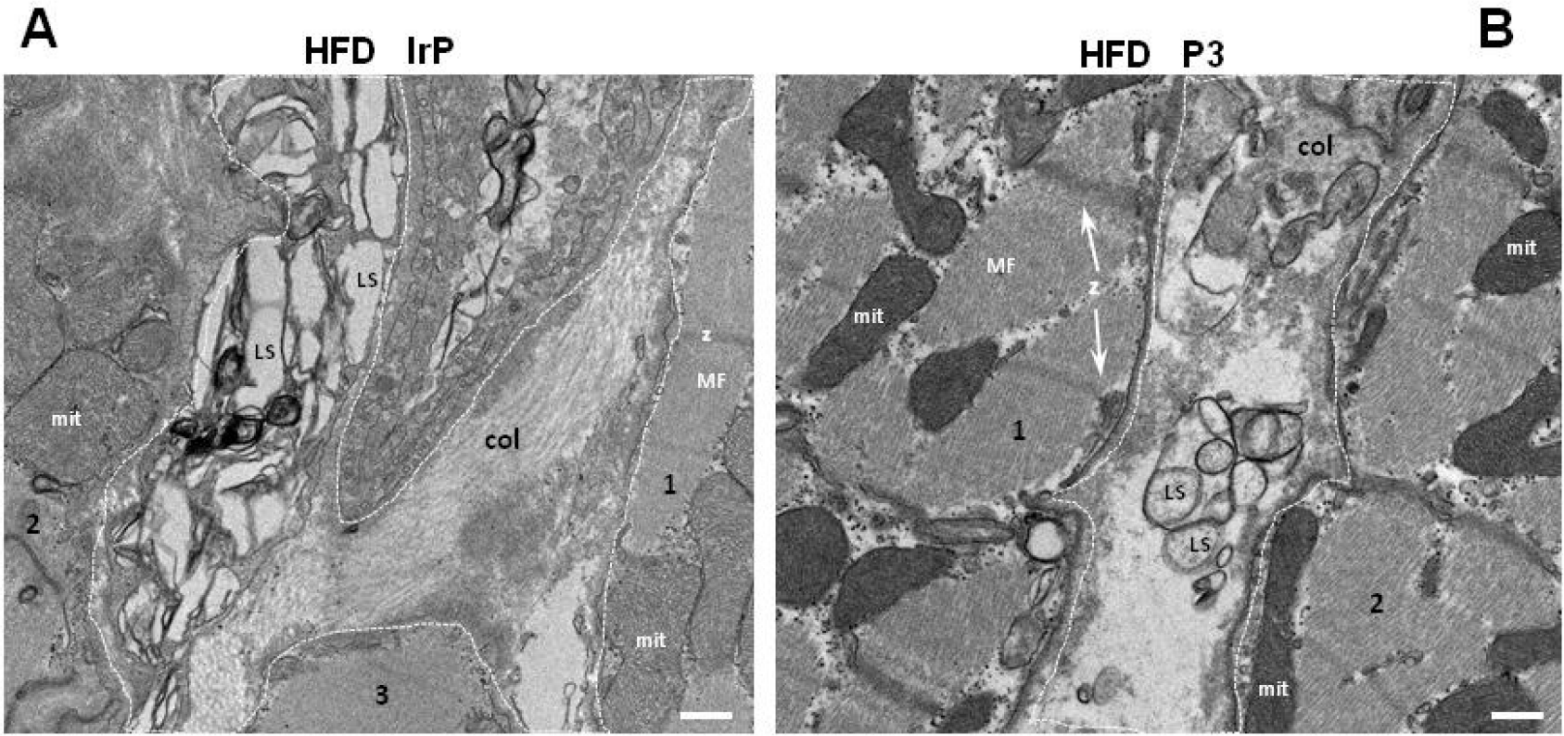
Anti-P3 Abs did not alter the HFD-induced extracellular plasticization in the heart. Representative transmission electron microscopy (TEM) images of cardiac intracellular and extracellular ultrastructure, showing the presence of lipid structures (LS) in the extracellular space (highlighted by dashed white points) in contact with (A) three cells (1–3) in HFD/IrP or (B) two cells (1 and 2) in HDP/P3 rabbit hearts. Abbreviations: Col, collagen; LS, lipid structures; mit, mitochondria; MF, myofibrils. Representative Z-discs (Z) are indicated. Numbers refer to different cells in the section. The scale bar is 400 nm.

TEM images showed the presence of extracellular lipid structures (LS) in both HFD/IrP hearts (Figure 8A) and HFD/P3 hearts (Figure 8B).

In line with calorimetric results, these results indicate that cardiac plasticization is caused by an extracellular phenomenon independent of intracellular cholesterol levels, in contrast to the other biophysical alterations in the ECM components. Due to their amphiphilic character, biomacromolecules are also known to interact with lipids via hydrophobic association, and lipids can act as efficient plasticizers.^63,65^ Previous studies from our group have shown that aggregated LDL favors a softness of tropoelastin.^25^ In addition, CE-enriched lipoproteins per se have been reported to produce an increase in the free volume of ECM macromolecules, causing a swelling and plasticization of the tissues.^66,67^ In line, the reduction of circulating levels of CE-enriched lipoprotein levels improved arterial stiffness in humans.^68^ On the basis of these results, we propose that the ECM plasticization of the softer phase in the heart is directly caused by the abundant extracellular CE-enriched lipoproteins generated by high-fat diets, and thus unchangeable by anti-P3 Abs. Thus, cardiac plasticization can be considered as a physicochemical phenomenon directly caused the accumulation of extracellular lipids.

Collagen is a main driver of progressive fibrosis.^69^ Here, we showed that excessive collagen deposition and cardiac fibrosis, as opposed to ECM plasticization, were associated with intracellular CE loading of cardiomyocytes because they were strongly inhibited in the heart of HFD/P3 rabbits. A crucial question is whether the relationship between intracellular cholesteryl ester loading and ECM deposition/fibrosis is direct or indirect and how intracellular CE loading could determine ECM deposition. Previous *in vitro* and *in vivo* studies from our group have consistently reported that intracellular CE loading upregulates LRP1 levels in vascular and cardiac cells.^13,14,18,28^ We showed the efficacy of anti-P3 Abs to reduce cardiac LRP1 levels in this rabbit model.^18^ LRP1 has been reported to regulate collagen synthesis in pleural mesothelial cells.^70^ Therefore, an alternative mechanism beyond CE transport that can explain the impact of anti-P3 Abs in ECM deposition is their capacity to modulate the levels of the LRP1 receptor. LRP1 could be a crucial player in the interplay between cardiomyocyte CE loading and cardiac fibrosis. Further studies are indeed required to gain insight into this crucial question.

## Conclusions

This is a multiapproach study that combines biophysical (FTIR and DSC) and imaging (optical and electron microscopy) techniques to evaluate the impact of hypercholesterolemia promoted by HFD on cardiac fibrosis, and the specific role of myocardial CE accumulation in ECM biophysical alterations. As summarized in the graphical abstract, the main finding of this study is that intracellular CE accumulation increases the level of ECM components such as collagen and PGs and the level of ECM hydration in the heart because these alterations are not detected in hypercholesterolemic rabbits in which myocardial CE accumulation has been specifically inhibited through LRP1-based immunotherapy. In contrast, this immunotherapy did not show efficacy to inhibit ECM plasticization induced by hypercholesterolemia in the hearts of rabbits, suggesting that this phenomenon is mainly related to the extracellular accumulation of CE-enriched lipoproteins.

The main strength of this study is having a model showing specificity and selectivity to target intracellular CE accumulation within the myocardium. The main limitation of this study is the impossibility of having cardiac functional data in this model or discerning which intracellular cholesteryl esters accumulate in which cell types and thus contribute to the alterations of the ECM. A further limitation is that the inflammatory mediators potentially produced by the myocardial cholesterol accumulation have not been evaluated, either in circulation or in the cardiovascular system.

Considering that intramyocardial CE accumulation in the heart is promoted by high-prevalence pathological situations, including ischemia,^12−14^ hypercholesterolemia,^16−18^ and obesity,^26^ all of them associated with dyslipemia, LRP1-based immunotherapy emerges as a valuable strategy for modulating not only cardiac sensitivity but also cardiac fibrosis in these groups of patients. However, additional and/or complementary strategies are required to avoid the pathological ECM plasticization associated with dyslipidemia.

## Experimental Section

### Study Design, Description of the Animal Model, and Collection of Cardiac Samples

Experimental procedures were approved by the Ethics Committee of Animal Experimentation of the Vall d’Hebron Institute of Research with registration number 46/17 and were performed in accordance with Spanish legislation as well as with the European Union directives (2010/63/EU). New Zealand white (NZW) rabbits were fed with (i) “standard chow” R-01 diet from Granja San Bernardo [17.3% protein, 16.7% fiber, and 3% fat] or a high-fat diet (HFD) of TD.140140 (1% cholesterol) (Harlan) for 1 month. Animals were acclimated for 1 week before the first immunization and then immunized every 21 days with a primary injection followed by four booster doses (R1–R4), with an irrelevant peptide (IrP group; *n* = 10) or P3 (P3 group; *n* = 10) conjugated to the carrier. The four doses of the IrP or P3 antigen conjugated with keyhole limpet hemocyanin (KLH) were administered subcutaneously (138 μg/kg, maximum volume of 150 μL). For the first immunization, IrP or P3-KLH peptides were emulsified in Complete Freund’s Adjuvant; the rest of the immunizations were performed using IrP or P3-KLH conjugated in Incomplete Freund’s Adjuvant (both from Sigma-Aldrich). During the immunization period, the animals were fed a standard chow diet. Starting at the R4 time point, IrP and P3-immunized rabbits were randomly divided into a standard chow-fed group or a HFD-fed group. Ten rabbits (*n* = 5 each of IrP-injected or P3-injected) continued to be fed a standard chow diet, whereas 10 rabbits (*n* = 5 each of IrP-injected or P3-injected) received a HFD for 30 days. Thus, four groups were analyzed: (i) standard chow, IrP-immunized (chow/IrP); (ii) standard chow, P3-immunized (chow/P3); (iii) HFD, IrP-immunized (HFD/IrP); and (iv) HFD, P3-immunized (HFD/P3). At pre- and postdiet time points, animals were weighed, and the serum levels of specific anti-P3 Abs were determined by an enzyme-linked immunosorbent assay (ELISA).^18,31^ At the end of the study, animals were euthanized, and the hearts were aseptically removed and excised to collect midmyocardium transmural left ventricular samples that were immediately frozen at −80 °C for molecular, immunohistochemical, imaging, and biophysical characterization.

### Peptide Synthesis and Conjugation

The P3 peptide used to immunize rabbits contained the sequence GDNDSEDNSDEENC, which corresponds to amino acids 1127–1140 located in LRP1 cluster II (domain CR9).^29^ The P3 sequence corresponds to an area with a high degree of homology between human and rabbit LRP1, with the difference that the asparagine (N) (1135) in humans is replaced by a serine (S) in the rabbit protein. In addition, amino acid 1140 in the rabbit sequence (GDNDCEDN**S**DEEN**C**) was replaced by C to achieve greater peptide immunogenic effectiveness. The irrelevant peptide (IrP) has the same sequence as P3 but with amino acids in the d-enantiomer configuration. Both peptides were >95% pure as determined by high-performance liquid chromatography (HPLC) analysis (Figure S8).

Both peptides were synthesized by the Laboratory of Proteomics & Protein Chemistry, Department of Experimental & Health Sciences, Pompeu Fabra University, by the solid-phase method using a Prelude peptide synthesizer (Protein Technologies, Inc.). Peptides were purified by HPLC (Waters 600) using ultraviolet detection at 254 nm (Waters 2487) and characterized by mass spectrometry (Applied Biosystems 4700 Proteomics Analyzer).

Peptides were conjugated to the transporter molecule keyhole limpet hemocyanin (KLH) for immunizations and with bovine serum albumin (BSA) for ELISAs. The conjugation of the peptide to KLH and BSA (Sigma, St. Louis, MO) was performed as previously described.^29^ Peptide-KLH conjugates were used for rabbit immunization and peptide-BSA conjugates for the substrate in the ELISA to detect specific anti-P3 Abs in the rabbit serum, as previously described.^18,31^

### Gene Expression Analyses by RT-PCR

Total RNA (1 μg) was used for cDNA synthesis according to the protocol provided with the High-Capacity cDNA Reverse Transcription kit (Applied Biosystems). cDNA was stored at −20 °C until its use. Gene expression analyses of 3-hydroxy-3-methyl-glutaryl-coenzyme A reductase (*Hmg-CoA reductase*, Oc06714507_m1), cholesterol 7α-hydroxylase (*Cyp7A1*, Oc04250258_m1), and low-density lipoprotein receptor (*Ldlr*, Oc03396245_m1) were performed in rabbit hearts by quantitative real time reverse transcriptase-polymerase chain reaction (q-RT-PCR) in the Applied Biosystems 7300 Real Time PCR System (Applied Biosystems, Foster City, CA). *18srRNA* (4319413E) was used as a housekeeping gene. The mRNA expression levels were measured in triplicate. The threshold cycle (Ct) values were normalized to the housekeeping gene.

### Immunohistochemistry

Myocardial collagen was immunohistochemically assessed by Sirius red staining. Four images of each sample were taken with a light microscope for polarization (DMi8, Leica, Wetzlar, Germany) and quantified with ImageJ version 1.50i. From the total area of the image and using the threshold tool, the area stained by the marker was selected and the percentage of staining was calculated with the Analyze option. The results expressed are the mean of the four quantifications that were carried out.

### Transmission Electron Microscopy (TEM)

Rabbits were anesthetized, and the hearts were removed and rapidly frozen and embedded in OCT. In some experiments, small 2 mm pieces were cut, and the tissue was fixed in 3% glutaraldehyde with 0.1 M phosphate buffer overnight. Postfixation was performed for 1 h at 4 °C in 1% OsO_4_ phosphate buffer, and after extensive washing, samples were dehydrated, embedded in Spurr, and sectioned using a Leica ultramicrotome (Leica Microsystems). Ultrathin sections (50–70 nm) were stained with 2% uranyl acetate for 10 min and with a lead staining solution for 5 min and then observed using a model JEOL JEM-1010 transmission electron microscope, fitted with a Gatan Orius SC1000 (model 832) digital camera, at the Unit of Electron Microscopy, Scientific and Technological Centers of the University of Barcelona, School of Medicine and Health Sciences (Barcelona, Spain).

### Vibrational Characterization by Fourier Transform Infrared Spectroscopy in the Attenuated Total Reflectance Mode (FTIR-ATR)

FTIR-ATR spectra of the freeze-dried tissues were acquired using a Nicolet 5700 FTIR spectrometer (Thermo Fisher Scientific, Waltham, MA) equipped with an ATR device with a KBr beam splitter and a MCT/B detector as previously described.^35,37,39^ The ATR accessory used was a Smart Orbit instrument with a type IIA diamond crystal (refractive index 2.4). Freeze-dried samples (1 mg) were directly deposited on the entire active surface of the crystal and gently pressed with a Teflon tip to ensure good contact. For each sample, 80 interferograms were recorded in the range of 4000–450 cm^–1^, co-added, and Fourier transformed to generate an average spectrum of the sample with a nominal resolution between overlapping bands of 1 cm^–1^ using Omnic 8.0 (Thermo Fisher Scientific). A single-beam background spectrum was collected from the clean diamond crystal before each experiment, and this background was subtracted from the average spectrum. Spectra were then subjected to ATR and baseline corrections and normalized using the maximum of the amide II peak. Second derivatives were used to enhance the chemical information present in overlapping infrared absorption bands of spectra. Mean spectra from each category of rabbit hearts were generated to obtain a clearer representation, and main bands were identified according to literature data (Table S1). For quantitative analysis, the area-of-interest bands were computed from each individual spectrum, and the appropriate area ratio proportional to the amount of the different components was generated.

### Thermal Analysis by Differential Scanning Calorimetry (DSC)

Calorimetric analyses of fresh or freeze-dried samples were performed using a DSC Pyris calorimeter (PerkinElmer, Waltham, MA). The calorimeter was calibrated using Hg and In as standards, resulting in a temperature accuracy of 0.1 °C and an enthalpy accuracy of 0.2 J/g. Fresh samples, 5–10 mg in weight, were placed in hermetic aluminum pans and equilibrated at the initial temperature for 5 min before cooling to −100 °C at a rate of 10 °C/min. The thermograms were then recorded during the heating at a rate of 10 °C/min until the temperature reached 90 °C. Once DSC measurements were performed, the pans were reweighed to check whether they had been correctly sealed. The sample pans were then pierced and dried to a constant mass at 105 °C for 14 h to determine the sample dry mass. Freeze-dried rabbit hearts, 5–6 mg in weight, were placed in nonhermetic aluminum pans and equilibrated at the initial temperature for 5 min before cooling to −100 °C at a rate of 20 °C/min. The thermograms were recorded during the first heating at a rate of 20 °C/min until the temperature reached 150 °C. The pans were reweighed after this first stage of dehydration, and the thermograms were recorded during the second heating at a rate of 20 °C/min until the temperature reached 250 °C.

### Statistical Analysis

Data analyses were performed with the statistical software R (www.r-project.org). Continuous variables were plotted and expressed as the mean ± standard deviation (SD). After the conditions for application had been checked (normality and variance homogeneity), one-way analysis of variance followed by Tukey’s post hoc test was used to compare groups with *n* > 5. A nonparametric Mann–Whitney U test was used for groups with *n* < 5. Differences were considered to be statistically significant when *p* < 0.05. A multivariate statistical analysis of the FTIR spectra of rabbit hearts has been performed using PCA in the [3700–2400 cm^–1^]∪[1800–630 cm^–1^] zone, using The Unscrambler X 10.5 (Camo Software).

## Data Availability

Data that support the findings of this study are available from the corresponding author upon reasonable request.

## Supporting Information Available

Seven figures and one table with additional information about mean FTIR-ATR spectra and second-derivative FTIR spectra of the four groups of rabbit hearts in selected zones of interest, principal component analysis of the FTIR spectra of rabbit hearts, and one FTIR table of the absorption bands in rabbit hearts and associated references (PDF)

## Abbreviations

Abs: antibodies
CE: cholesteryl esters
DSC: differential scanning calorimetry
ECM: extracellular matrix
FTIR-ATR: Fourier transform infrared spectroscopy in the attenuated total reflectance mode
HFD: high-fat diet
LDL: low-density lipoprotein
HMG-CoAR: 3-hydroxy-3-methyl-glutaryl-coenzyme A reductase
LDLR: low-density lipoprotein receptor
LRP1: low-density lipoprotein receptor-related protein 1
SERCA2: sarco(endo)plasmic reticulum calcium ATPase-2
VLDL: very-low-density lipoproteins
TEM: transmission electron microscopy
TG: triglyceride
