# Supplementary material for: Inhibitory Effects of LRP1-Based Immunotherapy on Cardiac Extracellular Matrix Biophysical Alterations Induced by Hypercholesterolemia
Source: J Med Chem. 2023 Apr 28;66(9):6251–62. doi: 10.1021/acs.jmedchem.2c02103 (PMC10184115; doi:10.1021/acs.jmedchem.2c02103)
Supplement: Supplementary file 1 — jm2c02103_si_001.pdf [file jm2c02103_si_001.pdf]

Inhibitory effects of LRP1-based immunotherapy on cardiac extracellular matrix biophysical alterations induced by hypercholesterolemia

Valerie Samouillan[1], Eduardo Garcia[2,3], Aleyda Benitez-Amaro[2,3], Maria Teresa La Chica Lhoëst[2,3], Jany Dandurand[1], Virginia Actis Dato[4,5], Jose Maria Guerra[6,7], Joan Carles Escolà-Gil[8], Gustavo Chiabrando[9], Carlos Enrich[10], and Vicenta Llorente-Cortes*[2,3,7]

[1]CIRIMAT, Université de Toulouse, Université Paul Sabatier, Equipe PHYPOL, 31062 Toulouse, France

[2] Biomedical Research Institute Sant Pau (IIB SANTPAU), Universitat Autonoma de Barcelona, 08041 Barcelona, Spain

[3]Institute of Biomedical Research of Barcelona (IIBB)-Spanish National Research Council (CSIC), 08036 Barcelona, Spain

[4]Departamento de Bioquímica Clínica, Facultad de Ciencias Químicas, Universidad Nacional de Córdoba, X5000HUA Córdoba, Argentina

[5] Consejo Nacional de Investigaciones Científicas y Técnicas (CONICET), Centro de Investigaciones en Bioquímica Clínica e Inmunología (CIBICI), Godoy Cruz 2290 Buenos Aires, Argentina

[6]Department of Cardiology, Hospital de la Santa Creu i Sant Pau, Biomedical Research Institute Sant Pau (IIB-SANTPAU), Universitat Autonoma de Barcelona, 08025 Barcelona, Spain

[7]CIBERCV, Institute of Health Carlos III, 28029 Madrid, Spain

[8]Metabolic Basis of Cardiovascular Risk, Biomedical Research Institute Sant Pau (IIB Sant Pau), 08041 Barcelona. CIBER de Diabetes y enfermedades Metabólicas Asociadas (CIBERDEM), 28029 Madrid, Spain

[9]Instituto Universitario de Ciencias Biomédicas de Córdoba (IUCBC), Centro de Investigación en Medicina Translacional Severo R. Amuchástegui (CIMETSA); G.V. al Instituto de Investigación Médica Mercedes y Martín Ferreyra (INIMEC-CONICET-UNC), X5016KEJ Córdoba, Argentina

[10] Unitat de Biologia Cel·lular, Departament de Biomedicina, Facultat de Medicina i Ciències de la Salut, Universitat de Barcelona, 08036 Barcelona, Spain; Centre de Recerca Biomèdica CELLEX, Institut d'Investigacions Biomèdiques August Pi i Sunyer (IDIBAPS), 08036 Barcelona, Spain.

Corresponding author: Vicenta Llorente Cortes. Institute of Biomedical Research of Barcelona (IIBB)-Spanish National Research Council (CSIC), Biomedical Research Institute Sant Pau (IIB-SANTPAU), Barcelona, Spain. ORCID: 0000-0001-8591-7632. Email: Vicenta.llorente@iibb.csic.es; cllorente@santpau.cat

Supporting Information_Manuscript ID: jm-2022-02103c.R2

Table of Contents

Figure S1. Normalized mean FTIR-ATR spectra of rabbit hearts in the [3600-2800 cm^{-1}] (A) and the [1800-700 cm^{-1}] (B) zone with assignment of major bands

Figure S2. Infrared analysis reflects the massive CE/PL accumulation in HFD IrP rabbit hearts

Figure S3. Expression levels of HMGCoAR and LDLR in the heart of rabbits

Figure S4. PCA analysis of the FTIR-ATR spectra of rabbits hearts in the [3700-2400 cm-1]

$\cup$ [1800-630cm^{-}1] zone showing the discrimination of HFD IrP rabbit hearts

Figure S5. Second derivative infrared mean spectra of rabbit hearts in the [1300-1200 cm-1] (Amide III) zone

Figure S6. Second derivative infrared mean spectra of rabbit hearts in the [1000-900 cm^{-1}] zone.

Figure S7. Second derivative infrared mean spectra of rabbit hearts in the Amide I zone.

Figure S8. HPLC chromatograms showing that peptides used for immunization are > 95% pure

Table S1. FTIR absorption bands of rabbit hearts

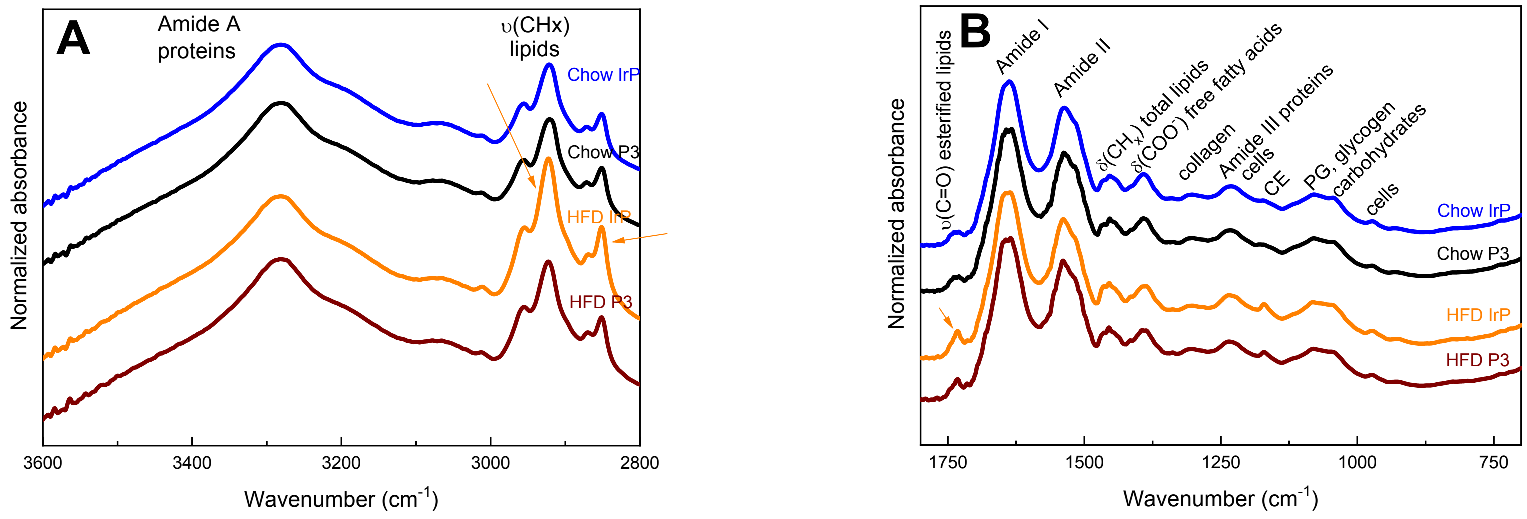

Figure S1: Normalized mean FTIR-ATR spectra of rabbit hearts in the [3600-2800 cm^{-1}] (**A**) and the [1800-700 cm^{-1}] (**B**) zone with assignment of major bands. Arrows in **A**) indicate the increase of the νas(CH_2) and νs(CH_2) bands mainly associated with total lipids for HFD IrP rabbit hearts; arrows in **B**) indicate the increase of the ν(C=O) band of the ester carbonyl groups associated with phospholipids and triglycerides for HFD IrP rabbit hearts.

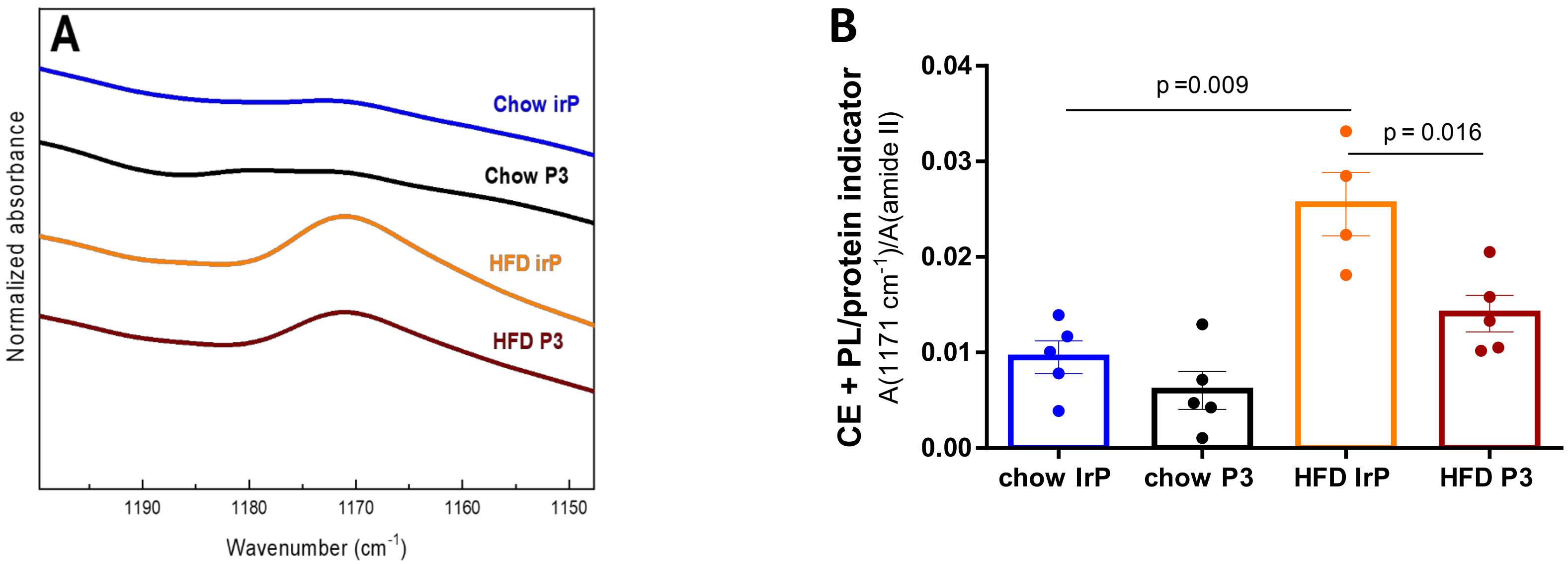

Figure S2. Infrared analysis reflects the massive CE/PL accumulation in HFD IrP rabbit hearts. (**A**) Normalized mean FTIR-ATR spectra of rabbit hearts in the zone of cholesteryl esters and phospholipids mixed absorptions. (**B**) Bar graphs showing the results from spectra quantitative analysis for associated components. n = 5/group. Results are shown as mean ± SD. Statistical significance was determined by Mann-Whitney U nonparametric test. The high dispersion of points may be related to the low intensity of certain bands and to the high cell variability between closed layers and closed heart zones previously reported in these complex cardiac tissues.[42, 43]

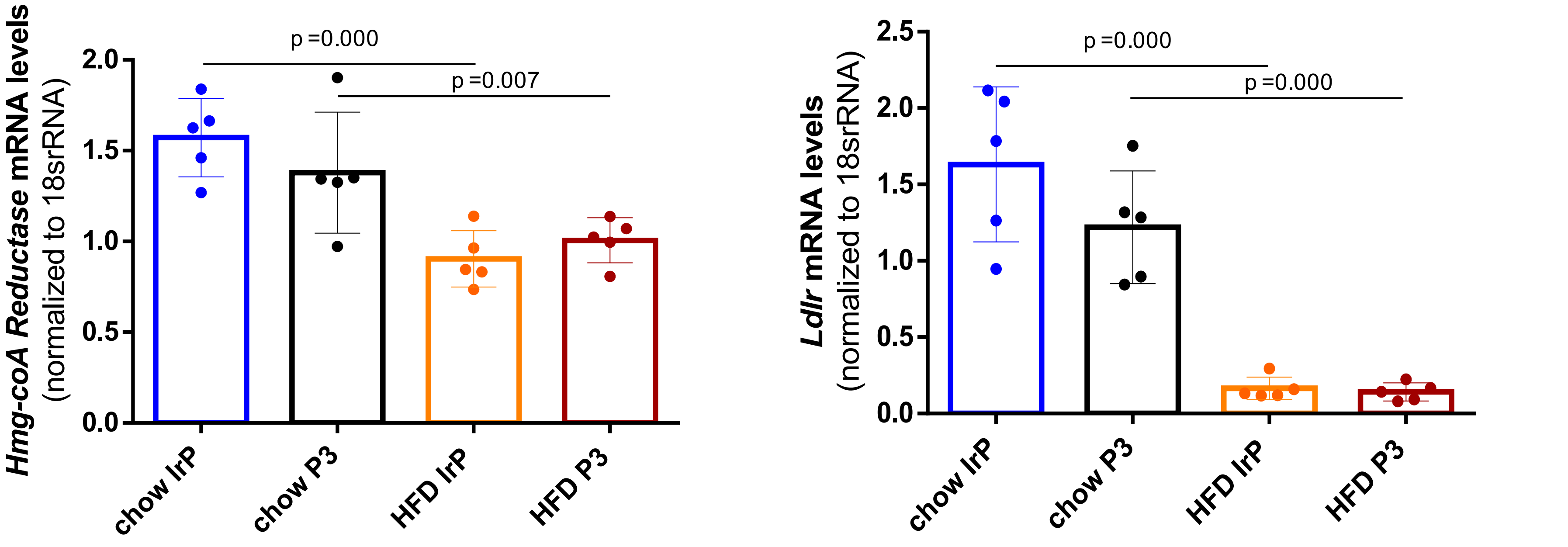

Figure S3. Expression levels of HMGCoAR and LDLR in the heart of rabbits. *Hmg-coA Reductase* and *Ldlr* mRNA expression levels were measured in the heart of rabbits by real time PCR. n = 5/group. Results are shown as mean ± SD. Statistical significance was determined by Mann-Whitney U nonparametric test. The high dispersion of points may be related to the high cell variability between closed layers and closed heart zones previously reported in these complex cardiac tissues.[42, 43]

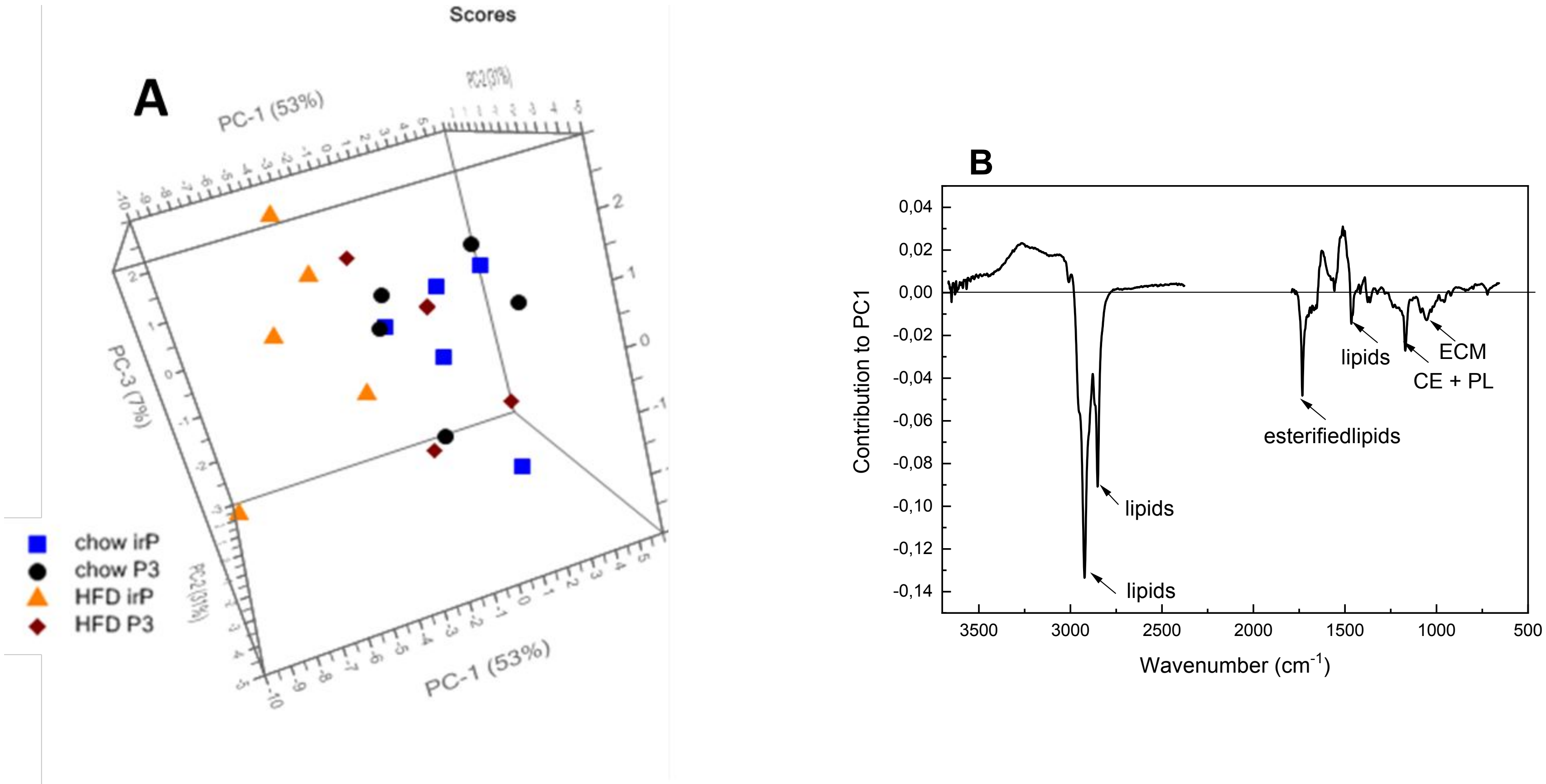

Figure S4. PCA analysis of the FTIR-ATR spectra of rabbits hearts in the [3700-2400 cm-1]∪[1800-630cm¯1] zone showing the discrimination of HFD IrP rabbit hearts. **(A)** 3D representation of each individual spectrum in the basis of the first three principal components PC1, PC2 and PC3. **(B)** Contribution of initial variables to the first principal component PC1. The high dispersion of points may be related to the low intensity of certain bands and to the high cell variability between closed layers and closed heart zones previously reported in these complex cardiac tissues.[42, 43]

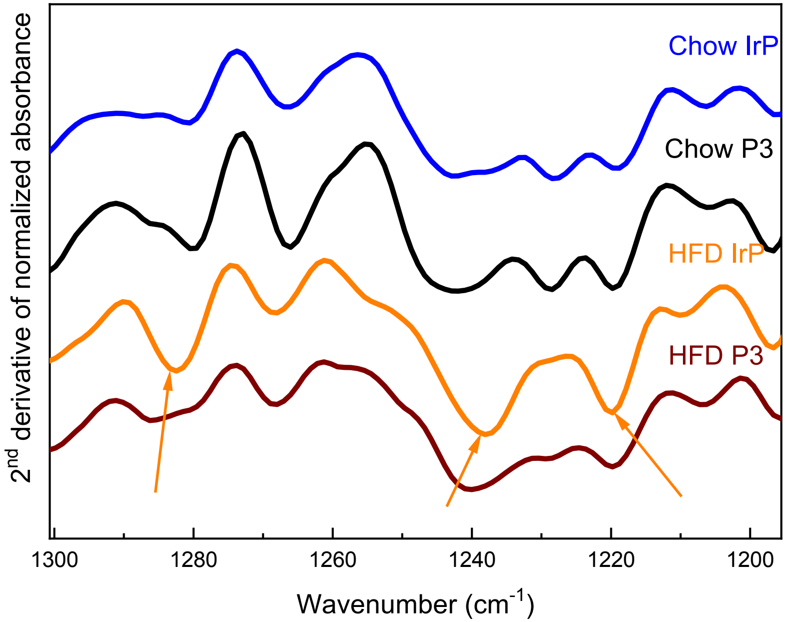

Figure S5. Second derivative infrared mean spectra of rabbit hearts in the [1300-1200 cm-1] (Amide III) zone. Arrows indicate the increase of the 1280, 1234 cm^{-1} bands (collagen), and 1226 cm^{-1} band (proteoglycans) for HFD IrP rabbit hearts.

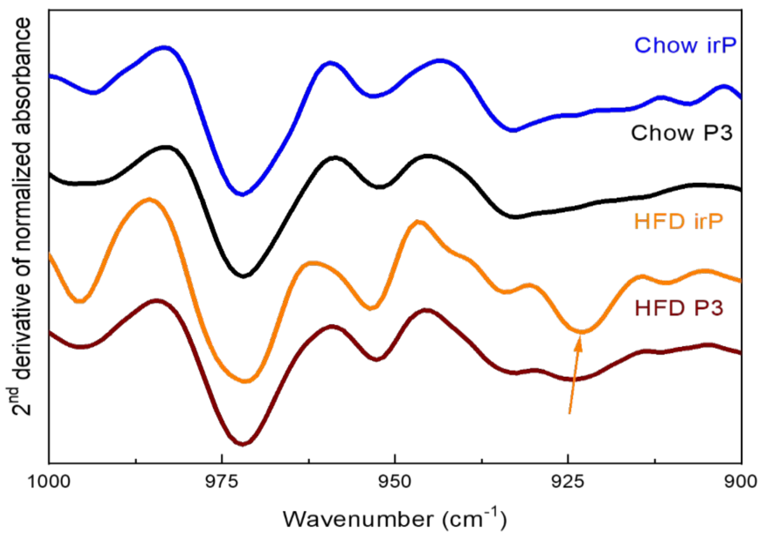

Figure S6. Second derivative infrared mean spectra of rabbit hearts in the [1000-900 cm^{-1}] zone. Arrow marks the increase of the 923 cm^{-1} band associated with Cα-C vibration for HFD IrP rabbit hearts.

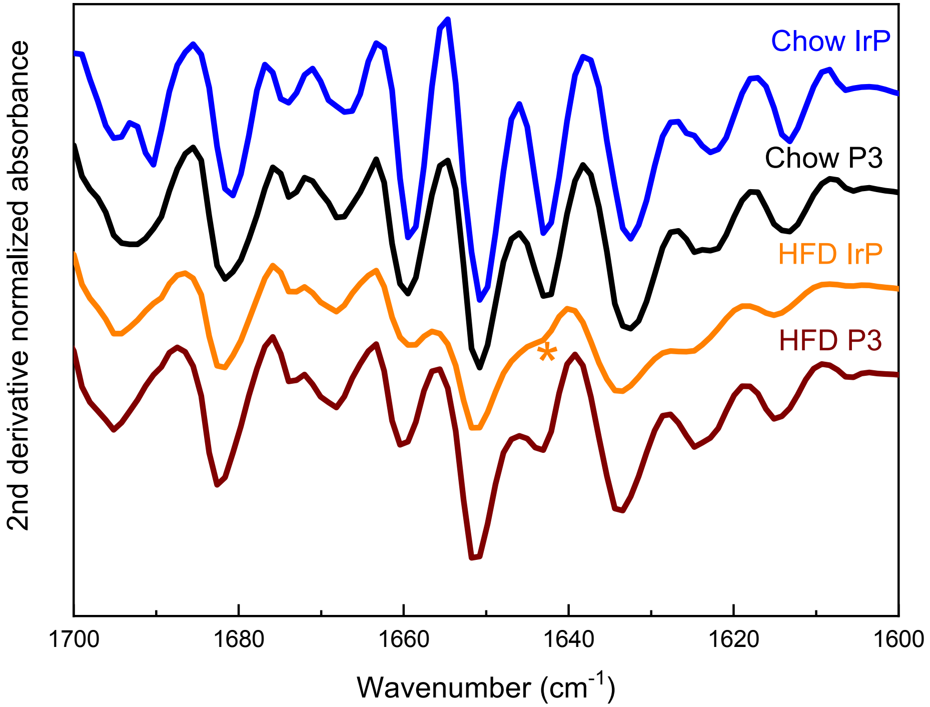

Figure S7. Second derivative infrared mean spectra of rabbit hearts in the Amide I zone. The different peaks in this zone indicate the composite character of the amide I band and can be assigned to the different secondary structures of proteins. Asterisk marks the decrease of the 1643 cm^{-1} component for HFD IrP rabbit hearts.

Purity

IrP

HPLC

Column HPLC Sunfire C18 (4.6 mm x 100 mm, 3.5 µm); Flow rate 1 mL/min; Gradient 0-100% B in 8 min (A= 0.045% TFA in H_2O and B 0.036% TFA in ACN).

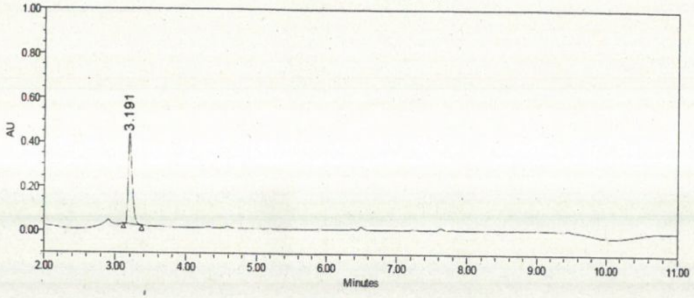

Result: Purity > 99.0%

Purity

P3

HPLC

Column HPLC Sunfire C18 (4.6 mm x 100 mm, 3.5 µm); Flow rate 1 mL/min; Gradient 0-100% B in 8 min (A= 0.045% TFA in H_2O and B 0.036% TFA in ACN).

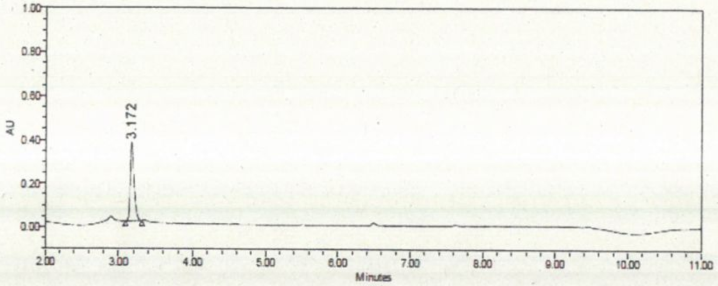

Result: Purity > 99.0%

Figure S8. HPLC chromatograms showing that peptides using for immunization are > 95% pure

**Band position (cm^{-1})**	**Assignment according to literature data** [1-11]
3282	Amide A, mainly the ν(N-H) mode of proteins with the contribution of the ν(O-H) stretching mode [1,2] of H_2O and polysaccharides
3072	ν(C-H) aromatic + amide B (C-H stretching and first overtone of amide II) in proteins, observed in collagen (Sionkowska 2011)
3012	ν(=CH) of unsaturated lipids, triglycerides, fatty acids<br>(mainly L phosphatidyethanolamine)
2955, 2922, 2871, 2851	$\nu_{as}(CH_3)$, $\nu_{as}(CH_2)$, $\nu_s(CH_3)$,$\nu_s(CH_2)$ of lipids and proteins
1743-1725	ν(C=O) of the ester carbonyl groups of phospholipids and triglycerides
1712	ν(C=O) of the nucleoside side of nucleic acids and free fatty acids
1644-1636	Amide I ν(C=O) of proteins – multi-component band sensitive to secondary structures of proteins
1538	Amide II ν(C-N), δ(N-H) of proteins
1515	Tyrosine band
1463, 1456	$\delta(CH_2)$ scissoring, $\delta(CH_3)$ bending of lipids and proteins
1392	νs(COO^-) of free amino acids, fatty acids, $\delta(CH_3)$
1337	$\delta(CH_2)$ wagging of the proline chain in collagen
1302<br>1280<br>1250-1220<br>1246-1235<br>1226	Specific to myofibers<br>Collagen amide III<br>Overlapping bands of amide III: δplan(N-H) and ν(C-N) of proteins<br>$\nu_{as}(PO_2^-)$ of phospholipids and nucleic acids<br>$\nu(SO_4^{2-})$ of proteoglycans
1200-1000<br><br>1171<br>1120<br>1080<br>1044	Overlapping bands of ν(C-O), ν(C-C), ν(C-OH), ν(C-O-C) of proteins, oligosaccharides, glycolipids, and $\nu_s(PO_2^-)$ of phospholipids and nucleic acids<br>ν_{as}(CO-O-C) of cholesterol esters, phospholipids<br>ν(C-O) lactate, polysaccharides<br>ν(C-O-C) of collagen, glycogen, oligosaccharides, glycolipids, and proteoglycans<br>ν_s(CO-O-C) of carbohydrates residues and polysaccharides
972	Phosphorylated proteins, phospholipids, and nucleic acids
922-930	υ(Cα-C)characteristic of α helices, υ(C-C) of proline and hydroxyproline ring, and Z-DNA
800	δ(C-H) bending out of plane of cholesterol and cholesterol ester

Table S1: FTIR absorption bands of rabbit hearts.

REFERENCES

(1) Staniszewska, E.; Malek, K.; Baranska, M. Rapid Approach to Analyze Biochemical Variation in Rat Organs by ATR FTIR Spectroscopy. *Spectrochim. Acta. A. Mol. Biomol. Spectrosc.* **2014**, *118*, 981–986. https://doi.org/10.1016/j.saa.2013.09.131.

(2) Stani, C.; Vaccari, L.; Mitri, E.; Birarda, G. FTIR Investigation of the Secondary Structure of Type I Collagen: New Insight into the Amide III Band. *Spectrochim. Acta - Part A Mol. Biomol. Spectrosc.* **2020**, *229*. https://doi.org/10.1016/J.SAA.2019.118006.

(3) Rehman, I. ur; Movasaghi, Z.; Rehman, S. *Vibrational Spectroscopy for Tissue Analysis*; CRC Press: Boca Raton, 2012. https://doi.org/10.1201/B12949/VIBRATIONAL-SPECTROSCOPY-TISSUE-ANALYSIS-IHTESHAM-UR-REHMAN-ZANYAR-MOVASAGHI-SHAZZA-REHMAN.

(4) Yang, T. T.; Weng, S. F.; Zheng, N.; Pan, Q. H.; Cao, H. L.; Liu, L.; Zhang, H. D.; Mu, D. W. Histopathology Mapping of Biochemical Changes in Myocardial Infarction by Fourier Transform Infrared Spectral Imaging. *Forensic Sci. Int.* **2011**, *207* (1–3), e34-9. https://doi.org/10.1016/j.forsciint.2010.12.005.

(5) Samouillan, V.; Revuelta-López, E.; Soler-Botija, C.; Dandurand, J.; Benitez-Amaro, A.; Nasarre, L.; de Gonzalo-Calvo, D.; Bayes-Genis, A.; Lacabanne, C.; Llorente-Cortés, V. Conformational and Thermal Characterization of Left Ventricle Remodeling Post-Myocardial Infarction. *Biochim. Biophys. Acta - Mol. Basis Dis.* **2017**, *1863* (6). https://doi.org/10.1016/j.bbadis.2017.02.025.

(6) Sionkowska, A.; Skopinska-Wisniewska, J.; Gawron, M.; Kozlowska, J.; Planecka, A. Chemical and Thermal Cross-Linking of Collagen and Elastin Hydrolysates. *Int. J. Biol. Macromol.* **2010**, *47* (4), 570–577. https://doi.org/10.1016/j.ijbiomac.2010.08.004.

(7) Byler, D. M.; Susi, H. Examination of the Secondary Structure of Proteins by Deconvolved FTIR Spectra. *Biopolymers* **1986**, *25* (3), 469–487. https://doi.org/10.1002/BIP.360250307.

(8) Popescu, M.-C.; Vasile, C.; Craciunescu, O. Structural Analysis of Some Soluble Elastins by Means of FT-IR and 2D IR Correlation Spectroscopy. *Biopolymers* **2010**, *93* (12), 1072–1084. https://doi.org/10.1002/bip.21524.

(9) Zohdi, V.; Wood, B. R.; Pearson, J. T.; Bambery, K. R.; Black, M. J. Evidence of Altered Biochemical Composition in the Hearts of Adult Intrauterine Growth-Restricted Rats. *Eur. J. Nutr.* **2013**, *52* (2), 749–758. https://doi.org/10.1007/s00394-012-0381-x.

(10) Wang, Q.; Sanad, W.; Miller, L. M.; Voigt, A.; Klingel, K.; Kandolf, R.; Stangl, K.; Baumann, G. Infrared Imaging of Compositional Changes in

Inflammatory Cardiomyopathy. *Vib. Spectrosc.* **2005**, *38* (1–2), 217–222. https://doi.org/10.1016/j.vibspec.2005.02.011.

(11) Hetman, Z. A.; Borchman, D. Concentration Dependent Cholesteryl-Ester and Wax-Ester Structural Relationships and Meibomian Gland Dysfunction. *Biochem. Biophys. Reports* **2020**, *21*, 100732. https://doi.org/10.1016/J.BBREP.2020.100732.
